# Hydrological Tour

**Published:** 1859-05

**Authors:** 


Hydrological Tour.—In giving some sketches of what we would term a hydrological tour, of which the quest after Thermal Springs will be the first part, we would remind our readers of quite a large class of what are termed, somewhat contradictorally but, at the same time, quite expressively, stay-at-home travelers. These persons, at any rate some of them, have read so fully and studied so carefully the geography, topography and appearances of a foreign land, and have made themselves so well acquainted with its characteristic features and its productive industry, and with the government and social state and manners of the people who inhabit it, that they can converse as if they had themselves been abroad, with the regular and known traveler, with a feeling of previous companionship and of sympathy on objects of common interest. John Randolph, of Roanoke, especially, in all that related to England, was of this class, long before he went abroad, which was late in his life. It would not be necessary to make this introduction to our sketches of hydrological travel, were we not desirous that our readers should not be deceived by the title, and be led to believe that we had accompanied, in early life, a Humboldt and a Bounpland to South America, or, in later times, an Atkinson to Siberia, or a Prince Napoleon to Iceland, or been a member of the United States Exploring Expedition, and thus visited the islands of the Pacific. We have, indeed, traveled and voyaged a little on our own separate and individual account, but more frequently and largely with the parties just mentioned, in the spirit, and with a great number of others not now to be named, by making their works our companions, for lack of ability to associate with the authors themselves.

It is not for the sake of contrasts, and as if to show that the most raging natural furnaces and the hottest water are to be found in the midst of perpetual snow and on the border of the Arctic circle, that we begin our thermo-hydrological tour with Iceland. It is an island of anomalies in a hygienic as well as in a geological, and, in reference to its inhabitants, ethnological point of view. With all the recognized elements for the development of phthisis, depending on the asperity of its climate, and the most trying vicissitudes of temperature and weather generally, and a stinted supply of alimentary substances, this disease is said to be very rarely met with in Iceland. This is one of the increasing number of exceptional facts to the common theory held respecting the climatic influences in the production and cure or alleviation of pulmonary consumption. It is time to begin a revision of the whole subject.

Some reference was made, in our January talk, to the celebrated erupting or spouting springs of Iceland, the Great and the Little Geyser, and the Stokkr, and of their analogy to volcanos. The relation which this class of springs has to earthquakes, has been noticed in different instances. A remarkable illustration of this connection was afforded in the Stokkr, in the New Geyser, and the Roaring Geyser, on the earthquake of 1789, in the circumstances of the former having only acquired its present size and character as a great erupting spring, and the latter having ceased to eject water, after that catastrophe. The Great Geyser, the largest and the most wonderful example of the pseudo-volcanic springs, resembles the volcano, in its eruptions of water and vapor being intermittent, and occurring at intervals of from 24 to 30 hours. The vapor is projected into the air with the rapidity of an arrow, and includes in its interior a column of water which rises in the air to the height of 80 to 90 feet. Soon the vapory clouds are dispersed and allow the column of water to be displayed with its innumerable jets, which are projected in a straight line upwards, and then spread out like the branches of a pine tree, and finally descend in the shape of a fine spray-like rain. The maximum temperature of the water at the bottom of the Great Geyser, at a depth of 72 feet, is 280·5° F.; the minimum, 251·5° F. The temperature of the surface is 135·2° F. when the bottom stood at the heat just mentioned. At the point of issue the water forms a chasm. This measures at the top 60 feet, and is 6 or 7 feet in diameter. The Stokkr, the greatest source of thermal eruption in Iceland, next to the Geyser, is only distant 140 yards from the latter. It is a funnel-shaped well 44½ feet deep, with a mouth of about 8 feet in diameter; its bore diminishing downwards until, at the depth of 27¼ feet, it is only 10 inches. The surface of the water, between the eruptions, is generally from 10 to 13 feet below that of the ground adjacent. The temperature at the bottom was 239° F., and is diminished with the distance upwards. At the height of fifteen feet it was 237 5° F., and at the surface the thermometer stood at 212° F., or the boiling point.

Besides these periodically eruptive springs, whose prototype is to be found in the two just described, there is another species of intermittent or thermal springs, which is not manifested by the sudden occurrence of detonations of vapor, and whose eruptions are not distinguished by sudden and rapid eruptions of boiling water. The Little Geyser, which belongs to the group of Reckum, furnishes one of the most remarkable examples of this species. Its water-spout rises, on a line between the vertical and the lateral, in jets to about the height of 30 or 40 feet. In the district of the Great Geyser, successive changes are taking place in many of the thermal springs, by which some have lost their Geyser character, and others have been entirely closed by successive silicate deposits. These are seen, in some instances, to break out in other places. Farther downwards, but above the thermal region, which is now in full activity, there are several reservoirs filled with hot water, in the depths of which the older Geyser mouths may still be seen through the continually increasing deposits of silica which have accumulated in the course of ages.

Analyses of the water of the Great Geyser, made by different persons, have detected, but in very minute quantities, carbonate of soda, sulphates of soda, potash and magnesia, chloride and sulphide of sodium, and carbonic acid. Bunsen and Sandbergei found carbonate of ammonia. The collective amount of all these solid substances is very small; it being only 1·26 in 1000 parts of the thermal water. Silex and alumina originally dissolved in the water, are precipitated by evaporation, and are not present in the water analyzed. The process by which all the mineral waters of Iceland obtain their constituents, appears to be in a series of very remarkable decompositions to which the palagonite is subject, under the influence of volcanic gases. The predominant element in this rock is silica, which is also the most conspicuous feature in the thermal waters of the island. Next to the silica, we find in this rock, in large proportion, sesquioxide of iron, then alumina, and after these, and in minute quantities, potash and soda.

The Gas Springs, as they are called by Krug von Nidda, consisting of emissions of sulphuretted hydrogen and steam, the fumeroles of Bunsen, are quite common in this part of Iceland. The soluble silicates are brought to the surface with the thermal water, giving rise, when it is left to evaporate freely, to the formation of siliceous sinter and opal. Hot mud-springs were seen by Henderson in the crater of the Krafla. In the Mynata lake there are several hot springs which are known to be such by the great clouds of vapor rising from various points on the surface of the water. Even at sea, as mentioned in a previous talk, hot spouting springs are seen as at Reykafiord in Isafiord, where four of them may be detected by the ascending steam, and one *huer* or boiling spring at the surface of the water. In the valleys in which lakes Apavatic and Tingvalla are situated, there are at least one hundred hot springs, resembling those of Hawkadal, in the Great Geyser district, and, like these, they are divisible into water and gas springs, many of which are periodical.

On the western side of Iceland, on a long, narrow tongue of land, stretching far out into the sea, there is a line of mineral springs different from the greater number of those dispersed on the trachyte, a palagonite region on the eastern side of the island, or in that of Hekla. They have a comparatively lower temperature, are rich in carbonic acid, both in a free and combined state, such as, in the latter cases, in the carbonates of soda and lime more particularly; and with these are found chloride of sodium and sulphate of soda. On the other hand, silica and sulphuretted hydrogen occur only in extremely minute quantities. These are the Beer Springs referred to by Bunsen. Numerous hot springs are given out from the southern valleys of the island.

The most noted near the southern coast are the hot springs of Reikum, to the north of a wide estuary, or almost lake, of the River Elvas. The Reikum are intermittent erupting springs, although there are but two of them which throw up the water to a considerable height, or with any regularity. The temperature is 212° F. The chief of the Reikum group of springs is the little Geyser, which we must not confound with a spring to which Mackenzie has given the same name: situated about one hundred yards south of the Slokkr. If the earth around the springs is turned up, a steam constantly rises, and in some places the surface is covered with sulphur. Occasionally there was seen a slight efflorescence which tasted like alum. In the ground in this district a great number of boiling springs and steaming apertures make their appearance, and it requires no small degree of courage to walk among them, as it is impossible to say how thin the arch of clay may be which separates the traveler from the boiling abyss below. In many spots in this vicinity one can see the interesting process, described by Stanley, of the conversion of the material furnished by the soft red clay which abounds here, into hard stone, resembling jasper, which will strike fire from steel. In the valley is a large chasm filled with boiling water, on one side of which there is a spring which throws out its water in a perfectly horizontal direction, emitting at the same time a great quantity of vapor, and bellowing with a very disagreeable noise. At a short distance, again, from this spring, lies a large pool of turbid water, from which a considerable column of the same liquid is almost uninterruptedly thrown to the height of nearly twenty feet. In the same vicinity are springs ejecting hot water from the very bed of the river. The whole tract of Olfus, in which Beikum is situated, is much exposed to earthquakes. It would seem, indeed, to be on a line of subterranean communication between Cape Ruykearmies, in the southeast, to Hekla in the south, and thence to the volcano mountain, Yokuls or Jokuls, in the northeastern part of the island. Several vapor springs are also found about Reikum. Around the aperture of the largest of these springs is a bank of blue and yellow clay, on the surface of which a considerable quantity of pure sulphur appears, and in some places small streaks of alum are visible.

The hot springs of Reikiavik, the capital of Iceland, on the southwestern coast, are ten miles east of the town, in a volcanic district. Their temperature is 188° F. The water of the springs, mixing with that of the rivulet into which they flow, allows of every desired temperature, within a short space. Some large and deep pools of a tepid temperature are formed, in which, on a certain day in June, all the girls in the neighborhood bathe. We must hope that they go through this process at other times, although in a less ceremonial fashion. Mackenzie, taking a northern route from Reikiavik, along the western coast, visited the hot springs of Leira, the temperature of which is 138° F. On the road-side north from Leira and south of Huaneyre, and not far from the mouth of the River Huitaa, are hot springs, the water of which is 100° to 132° F. It is pure, and forms no incrustation. At the upper end of the Huitaa or White River we meet with the hot springs of Rickhalt or Reckiadal. On plunging the thermometer into those which could be approached with safety, it was seen to stand at 212° F. A little farther up the valley, there issues from the highest point of a rock, about ten feet high, in the middle of the river, a jet of boiling water several feet in height. In the middle of the rock there is a hole about two feet from its base; the water boils strongly; and again a similar phenomenon presents itself near the other end of this rock. Two miles farther up the valley there is an ancient bath, built six hundred years ago. Reversing the usual order of things, there is no cold water in the neighborhood of this bath, and hence they who intend to use it, wait till it is cooled down to the desired point.

Down the valley, and on the side opposite to that we have just described, and near to a group of cottages, are some thermal springs. In one place the vapor, which was 73° F., was received in a hut, and used as a vapor bath. Even at this reduced temperature, and in the month of July, it was probably twenty degrees higher than the medium of the daily temperature of the external air. The room was used both for a vapor bath, and for drying clothes. About a mile farther down, at the end of the valley, is the *Tungarhuer*, an assemblage of springs, described by Mackenzie to be "the most extraordinary perhaps in the world." One of these, called *Ar-Huer*, or the river spring, is unique of its kind. Two rocks, within a yard of each other, give issue to two springs, which give out their jets alternately, the one playing so soon as the other ceases; the interval of time between the two jets being about four minutes. Mackenzie called these ejections by the name of "*alternating Geyser.*" Another spectacle meets the eye on this spot, viz., a vein of steam rising from the river itself, caused by boiling water finding its way through the river-bed. Some distance up the Huitaa is the hot spring of Sidcumele, the water of which shows a temperature of 76° F. The springs of Staffhold are six in number. Between them and the town is a field of lignite.

Near an extinct volcano, that of Kystardal, and several caves of lava, the traveler comes to a series of acidulous, called by the natives Ale springs. Not many miles farther, and still on the coast, the traveler comes to Stappla, from which towers the stupendous Snefell Yokull or Great Snow Mountain. Space is not given us to describe the hot springs near the northern coast of Iceland. The first to be named are the hot springs of *Reikialaug*, three in number, which have respectively the temperature of 114° F., 100° F., and 124° F. The next are the hot springs of *Reikeiverf*, the most remarkable of which are three in number. A truly volcanic region, with its widely distributed lava, sulphur mines, fumeroles, but water and mud springs and geysers and a smoking lake, opens out to the traveler at no great distance southeast from Husavick. Here he encounters Lake Myvatu, which is forty miles in circumference, has a moderate depth of say from two to four fathoms. Hot fountains boil up in the middle of the lake, and give out steam which is seen at a great distance. Reikiahlid, a farmhouse built on the lava which was poured out between 1726 and 1730, is on the north side of Lake Myvatu, is truly in a thermal neighborhood, one of the most prominent proofs of which is a natural hot vapor bath, supplied from a crevice in the ground, of the temperature of 144° F. This bath is frequented by people from a distance. Near to this place are vast beds of sulphur, giving out sulphurous exhalations through holes and crevices. To the east rises the Sulphur Mountain with its numerous burning quagmire and issuing springs. Near Krabla is the Obsidian Mountain. Along the eastern coast, and then the southern to near Reikum, we no longer meet with thermal springs.

On the whole route from Reikavik to the Geysers, in a line northeast, is Mufel, near which are several hot springs. On the margin of a small lake, called Lungavalla, are hot springs, some of them geysers, the eruptions recurring at irregular intervals.

Although in this brief and compressed notice of the thermal springs and thermography of Iceland we may fail to interest our readers, we have at least the consolation of knowing that the details are not accessible in any text book or compilation to which they can turn; and, so far, our dottings cannot be called stale or commonplace. Our next journey will be to a more congenial region.

